# Standard set of patient-reported outcomes for personality disorder

**DOI:** 10.1007/s11136-021-02870-w

**Published:** 2021-06-02

**Authors:** Valentina Prevolnik Rupel, Beth Jagger, Luz Sousa Fialho, Lisa-Marie Chadderton, Timea Gintner, Anroud Arntz, Åse-Line Baltzersen, Julia Blazdell, Jan van Busschbach, Marika Cencelli, Andrew Chanen, Charlotte Delvaux, Fieke van Gorp, Lucie Langford, Brian McKenna, Paul Moran, Karla Pacheco, Carla Sharp, Wei Wang, Karen Wright, Mike J. Crawford

**Affiliations:** 1 Institute for Economic Research, Kardeljeva ploščad 17, 1000 Ljubljana, Slovenia; 2 International Consortium for Health Outcomes Measurement, Cambridge, USA; 3 University of Amsterdam, Amsterdam, The Netherlands; 4 Patient Representative, The Norwegian National Advisory Unit On Personality Psychiatry, Oslo, Norway; 5 The Institute of Mental Health, WLMHT Managed Clinical Network, Southall, UK; 6 Erasmus MC, Rotterdam, The Netherlands; 7 Patient Representative, London, UK; 8 The University of Melbourne, Melbourne, Australia; 9 Patient representative, Te gek!?, Ghent, Belgium; 10 Patient Representative, Ghent, Belgium; 11 University of Toronto, Toronto, Canada; 12 Auckland University of Technology, Auckland, New Zealand; 13 University of Bristol, Bristol, UK; 14 Tecnologico de Monterrey, Monterrey, Mexico; 15 University of Houston, Houston, USA; 16 Norwegian University of Science and Technology (NTNU), Trondheim, Norway; 17 University of Central Lancashire, Preston, UK; 18 Imperial College, London, UK; 19 Swinburne University of Technology, Melbourne, Australia; 20 Orygen, Melbourne, Australia

**Keywords:** ICHOM, Patient-reported outcomes, Personality disorder, Quality of life, Risk-adjustment variables, Delphi procedure

## Abstract

**Purpose:**

The purpose of the article is to present standard set of outcomes for people with personality disorder (PD), in order to facilitate patient outcome measurement worldwide.

**Methods:**

The International Consortium for Health Outcomes Measurement (ICHOM) gathered a multidisciplinary international working group, consisting of 16 experts, including clinicians, nurses, psychologists, methodologists and patient representatives, to develop a standard set of outcome measures for people with PD. The Delphi method was used to reach consensus on the scope of the set, outcome domains, outcome measures, case-mix variables and time points for measuring outcomes in service users. For each phase, a project team prepared materials based on systematic literature reviews and consultations with experts.

**Results:**

The working group decided to include PD, as defined by International Classification of Diseases 11th revision (ICD-11). Eleven core outcomes and three optional outcomes across four health domains (mental health, behaviour, functioning and recovery) were defined as those relevant for people with PD. Validated measures for the selected outcomes were selected, some covering more than one outcome. Case-mix variables were aligned to other ICHOM mental health standard sets and consisted of demographic factors and those related to the treatment that people received. The group recommended that most outcomes are measured at baseline and annually.

**Conclusion:**

The international minimum standard set of outcomes has the potential to improve clinical decision making through systematic measurement and comparability. This will be key in improving the standard of health care for people with PD across the world.

## Introduction

People with personality disorder (PD) have problems in functioning of aspects of self and interpersonal dysfunction which lead to emotional distress and impaired social function [1]. With onset early in life [2], high prevalence of over 5% of the general population and 50% in the outpatient psychiatric settings [3], it contributes to a substantial portion of health-care spending [4]. Most of the costs are incurred by inpatient and community mental health care and increased levels of unemployment and lost productivity among people with PD. A variety of psychological and psychosocial interventions have been shown to improve the mental health of people with PD [5–7]. The wide consensus is that the primary treatment for PD should be outpatient psychosocial therapy, with pharmacological treatment used mainly for the treatment of coexisting conditions. Further recommendation regarding the length and modality of treatments for each trait profile of PD is not clear, differs among countries and are often not in line with the latest research [8]. Compared to other common mental disorders, personality pathology is rarely tracked in routine clinical care. While many settings routinely assess the outcomes of people with depression and anxiety [9], outcome assessment in PD is rare and mostly refers to borderline personality disorders (BPD). In BPD, meta-analyses and reviews highlight the variety of outcomes utilized. Stoffers et al. [10] defined primary outcomes, which included overall BPD severity and BPD symptoms severity; and secondary outcomes, which included psychiatric comorbidity, general distress, global assessment of functioning, attrition/noncompliance with treatment, and adverse events. Lieb et al. [11] used all the outcomes from Stoffers et al. but added hospitalizations, emergency department visits, medication tolerability and side effects. Further outcomes measured in longer-term studies are social and vocational functioning, symptomatic remission and recovery from BPD [12].

In order to establish the value of each treatment for each service user, monitoring of health outcomes is essential. Value of treatment is defined as ‘the outcomes achieved relative to the costs’ [13]. For multiple reasons, measuring outcomes in the mental health is less common and more difficult than elsewhere, in spite of many available and validated health outcomes measures [14]. First of all, measurement precision of instruments might be lower compared to biomarkers. In addition, many instruments are time consuming, and clinicians might lack resources to implement them in busy clinical settings [14]. Also, there are many outcome measures available to measure each domain or symptom, making the results difficult to compare.

ICHOM was established to review the existing outcome measures that matter most to patients and to outline minimum standard sets of outcomes, measurement instruments, timepoints and risk adjustment factors for various conditions [15]. In 2018, ICHOM set out to cover some of the most prevalent mental health conditions. An international, multidisciplinary working group, led by ICHOM, was set up in the end of 2018. Our aim was to define the outcomes that matter most to persons with PD and prepare the standardized set of instruments to measure these outcomes.

## Methods

### The working group

The development of standard set for PD was initiated by ICHOM, which sets up a small project team (M.C., L.S.F., B.J., L.-M. C, T.G and V.P.R) and a wider working group. The wider working group consisted of 16 experts, including clinicians, nurses, patient representatives and experts in the area of outcome measurement. Working Group selection criteria defined by ICHOM were strictly followed; the members of the Working Group committed to active engagement and participation and were selected to cover the breadth of expertise needed to develop the content of the standard set—clinical expertise, PROMs expertise and health-care system evaluation expertise. The working group members came from Europe, North America, Latin America, Middle East, Australia and New Zealand, representing all regions of the world. Their work was coordinated and guided by the project team.

### Work process and decision making

**Fig. 1:**
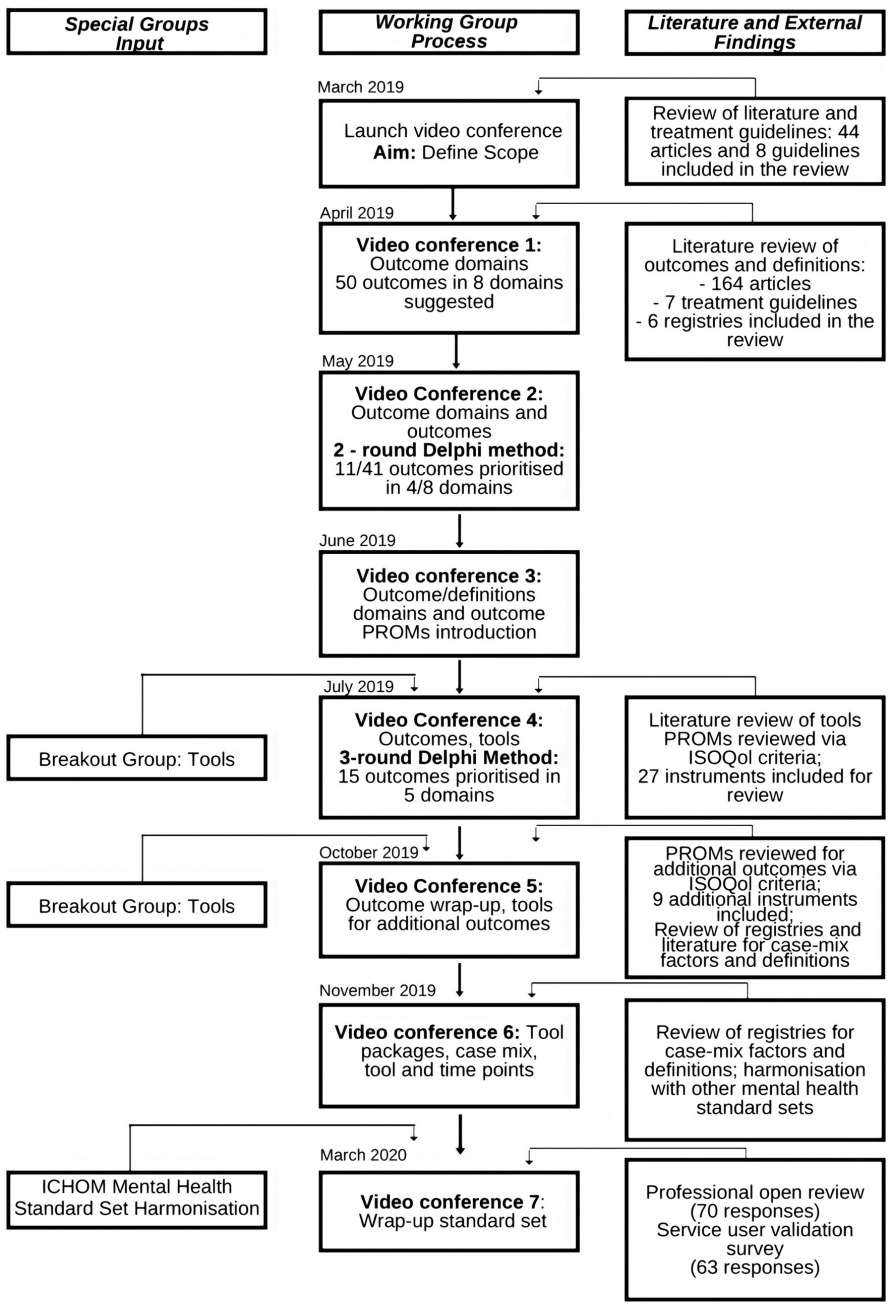
Process of the outcomes development

The working group convened via eight video calls from March 2019 to March 2020. Their work followed the Delphi process, previously modified and applied by ICHOM in the course of preparation of standard sets for a number of conditions [16–21]. A standard set of outcomes was developed through several phases (Fig. 1). Each teleconference had a previously determined goal, which was defined according to the issues that arose in the process of the development of the standard set. In line with the set goal, the project team prepared the research inputs based on the reviews of literature using common databases (PubMed, EMBASE, CINAHL, Medline, PsycINFO) and reviews of treatment guidelines and registries (e.g. Personality Disorders Registry Spain; Guideline on BPD: recognition and management, England; Guideline on Antisocial Personality Disorder: prevention and management, England; Guideline on Antisocial behaviour and conduct disorders in children and young persons: recognition and management, England; Guideline on Personality Disorders, Germany; National Outcomes and Case-mix Collection (NOCC), Australia; APA’s Mental health registry PsychPRO, USA; Mental Health Registry). Additionally, breakout groups were set up to discuss most relevant issues to decrease the complexity of the issues to be decided on at the working group calls.

Breakout groups were organized to discuss the issues of instrument selection and packages as well as to harmonize standard sets regarding outcome instruments, timepoints and case-mix variables across mental health working groups. At the teleconferences, gathered and analysed information, including proposals, was presented for group discussion. After each teleconference, the discussed content was organized into an online survey. It was emailed to working group members who were invited to vote on the issues discussed.

Content was included if 70% consensus was reached and excluded if less than 50% consensus was reached. Issues that remained inconclusive were further discussed and subjected to additional rounds of voting until a consensus was reached, following the rules from the previous sentence. At least an 80% of the group had to take part in a vote for it to be considered valid. A consensus had to be reached in four major decision areas: (1) scope: which conditions, population age and treatments should be included in the PD standard set, (2) outcome domains and outcomes in each domain, (3) instruments and instrument packages in each of the domains and (4) case-mix variables and timepoints.

To vote on outcomes, working group members discussed the long list of potentially relevant outcomes on the call and then voted online anonymously after reviewing the materials and minutes from the call. This was done using an online survey, where they were presented with each outcome and asked to rate the outcome on a scale from 1 to 9 (1 = not important, 9 = essential). Inclusion in the standard set required that a minimum of 80% of the consensus working group voted an item as “essential” (score of 7–9) in the first or second round Delphi vote. When consensus was not reached by voting, the item was discussed and revisited in the next videoconference and survey. Outcomes were excluded if a minimum of 80% of the consensus working group voted an item as “not recommended” (score 1–3). The consensus working group voted on all inconclusive outcomes in the final survey round, following ICHOM processes, in which the response options were simply “include” or “exclude”. In this final round, inclusion in the standard set required only 70% consensus. A similar process was used to reach consensus on recommended measures and risk adjustment factors.

### Definition of scope and selection of outcome domains and outcomes

**Fig. 2:**
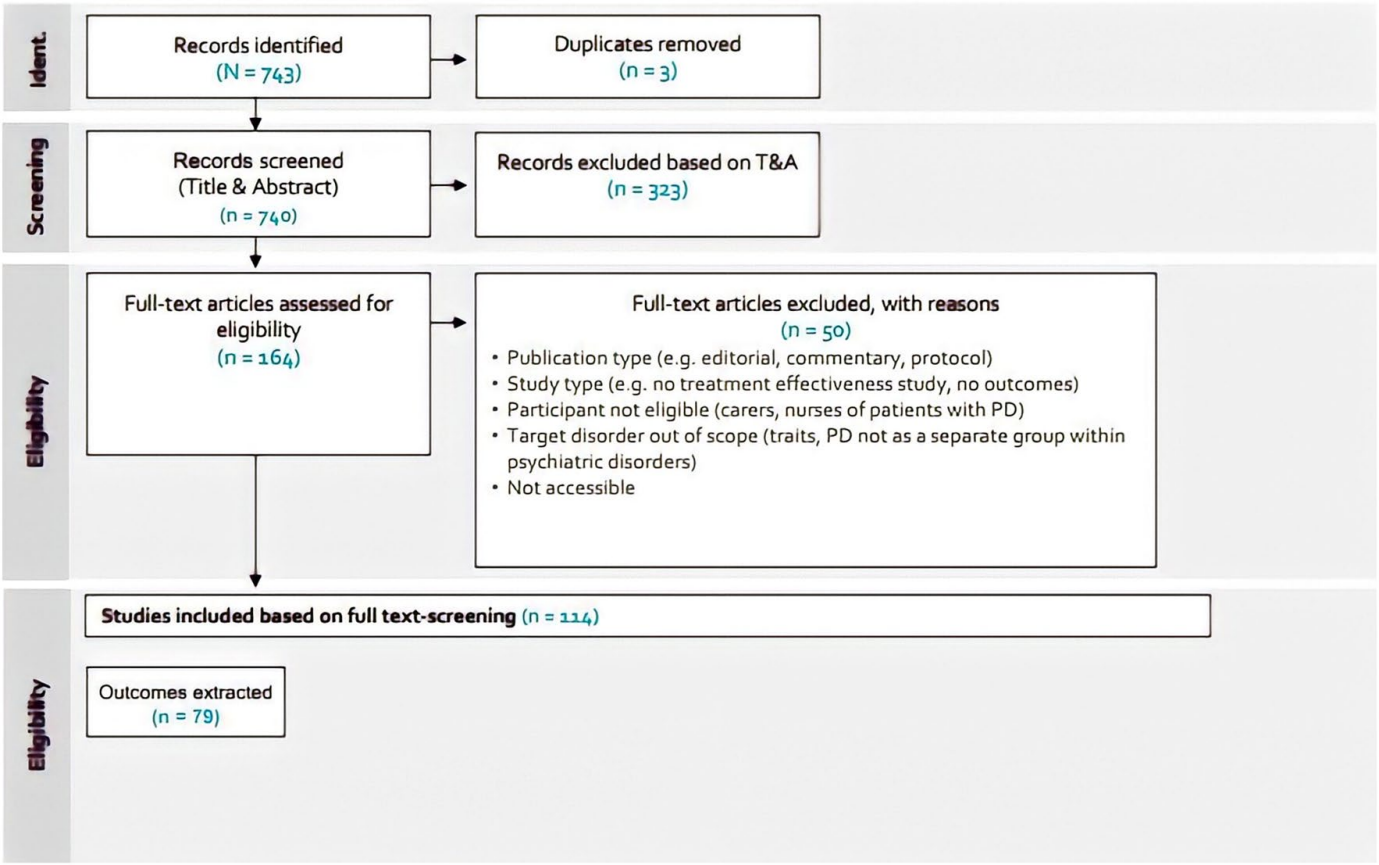
Search strategy and selection process for final inclusion of outcome domains considered for the final PD standard set

Preceding the launch call, a systematic literature review was performed in November 2018 to define the scope of the work. The following databases were searched: Medline and Embase in Ovid and CINAHL and Psychinfo in Ebsco. Out of 3270 articles identified, 49 were included in the scope definition. Due to the high number of hits the decision was taken to conduct all further searches in Medline at first and only extend the search to other databases if necessary. The following systematic literature search for outcome domains was conducted in Medline in March 2019 (Fig. 2). Additionally, treatment guidelines and registries were taken into account to develop the final definition of outcome domains and outcomes.

### Selection of outcome measures

The selection of outcome measures was based on the systematic literature review in Fig. 2. A total of 268 potentially relevant patient-reported outcome measures (PROMs) were screened with respect to (1) conceptual and measurement model, (2) evidence supporting psychometric properties, e.g. validity and reliability, (3) clinical utility, (4) feasibility of implementation (licensing fees – measures that need to be paid for were excluded, number of language translations, number of citations, and service user and administrative burden – length of the questionnaire and (5) harmonization with other mental health standard sets. Additional literature searches were conducted in PubMed for each measure undergoing screening. The measures that passed the initial screening by the project team of the 268 potentially relevant patient-reported outcome measures (PROMs) identified were then presented to the working group, alongside evidence supporting psychometric properties, e.g. validity and reliability. The working group discussed the issues around clinical utility, psychometric properties, feasibility of implementation and benchmarking potential during the working group call.

Following this discussion, the working group members voted anonymously on an online survey about which measure should capture which outcome individually. The decision to include or exclude a measure required 70% consensus, with a minimum of 80% participation from working group members.

To establish cross-cultural equivalence between the various countries, a list of case-mix variables was extracted from the registries and PD guidelines. Case-mix variables (Table 4) describe the context in which the outcomes are measured. To ensure high level of harmonization, previous ICHOM standard sets were reviewed for definition of demographic and socioeconomic variables.

### External validation by health professional and service user experts

In February 2020, ICHOM presented a draft recommended PD standard set, which was sent into open review process by professionals and into service user validation process. Any results securing an endorsement higher than 70% from the open review panel (service users) were accepted, while those receiving a lower endorsement went into further discussion with working group members.

Search term: (“personality disorder” [ti] OR “borderline personality disorder”[tiab] OR “schizotypal personality disorder”[tiab] OR “schizoid personality disorder”[tiab] OR “histrionic personality disorder”[tiab] OR “narcissistic personality disorder”[tiab] OR “paranoid personality disorder”[tiab] OR “avoidant personality disorder”[tiab] OR “antisocial personality disorder”[tiab] OR “dependent personality disorder”[tiab] OR “obsessive–compulsive personality disorder”[tiab] OR “Negative affectivity in personality disorder or personality difficulty”[tiab] OR “Detachment in personality disorder or personality difficulty”[tiab] OR “Dissociality in personality disorder or personality difficulty”[tiab] OR “Disinhibition in personality disorder or personality difficulty”[tiab] OR “Anankastia in personality disorder or personality difficulty”[tiab] OR “Borderline pattern”[tiab]) AND (meta-analysis [ti] OR review [ti]). Articles from 2009 on were included.

## Results

### Scope

The working group decided to include PD as defined by International Classification of Diseases 11th revision (ICD-11) [1]. Substance use-induced PD, PD due to organic causes including head injury, personality change/disorder secondary to other mental health condition, subthreshold personality dysfunction and personality difficulty were excluded from the scope of the project. The settings included primary care, inpatient and outpatient care, day hospital, community treatment, forensic mental health services, family care, and criminal justice care in a form of group as well as individual therapies. All psychotherapeutic and pharmacological treatments were voted within scope, except use of drugs for comorbid conditions. Recommendations were limited to adults and adolescents aged 13 years or above – for children aged 2–12 that there is not much literature on PDs and the outcomes measures used are different. The literature [22] suggests that PD begins in childhood and adolescence, and can be diagnosed in young people. For example, BPD is common among young people: the estimated prevalence is 1–3% in the community, rising to 11–22% in outpatients, and 33–49% in inpatients. BPD is one of the leading causes of disability-adjusted life years (DALYs) in young people among mental diseases and represents a substantial financial burden for the families of young people. The effectiveness of structured treatments for BPD in young people has been demonstrated.

### Outcome domains and measures

Based on the literature review, a list of 50 outcomes in eight outcome domains was proposed for voting. This list was later expanded and refined following the suggestions, discussion and three rounds of Delphi voting by working group members. The final list consists of 14 outcomes, grouped in four outcome domains [9]: (1) Mental health, (2) Behaviour, (3) Functioning and (4) Recovery. All the outcomes considered for the inclusion in the PD standard set are presented in Table 1.

**Table 1:** List of all outcomes proposed for voting to working group

Included outcomes	Excluded outcomes	
Emotional dysregulation	Anxiety	Mental well-being
Emotional distress	Capacity for empathy	Community participation
Suicide ideation and behaviour	Depression/low mood	Resilience
Self-harm	Dissociation	Self-compassion
Impulsivity	Emptiness	Self-esteem
Global functioning	Guilt	Self-efficacy
Interpersonal functioning	Hopelessness	Caregiver-youth relationship
Social functioning	Obsessive rigidity	Criminal activity
Sense of belonging	Personality organization/pathology/temperament	Family burden
Self-care	Suicide	Family mental health
Health-related quality of life	Substance misuse	Stigma
Identity disturbance	Abuse of others (harming loved ones)	Time to treatment
Aggression	Pain	Time to diagnosis
Severity of personality disorder	Mortality	Use of health services
Coping with Past Experiences of Trauma*	Sleep	Hospital admission
	Self-awareness	Cost of treatment and care
	Sense of hope	Use of other services
	Satisfaction with services	Patient-reported experience

*No adequate instruments for ‘Coping with Past Experiences of Trauma’ were identified

A comprehensive literature review was performed for each of the outcomes in order to identify the instruments within the defined scope of the standard set. A total of 268 instruments identified were screened and reduced to 13 instruments (Table 2). A breakout group was established to help ensure that the measures were harmonized to the highest possible degree among mental health standard sets. As there were four mental health sets in development simultaneously and all of them included “Functioning” and “Health-Related Quality of Life", the same instruments to cover the same domain across the mental health sets were used. Members of the group also expressed a preference for measures that were appropriate for both adolescents and adults in order to enable tracking the mental health outcomes during this period.

**Table 2:** Presentation of instruments covering the selected outcomes

Instrument	No. of items	Data reported	No. of languages	Age range covered	Time to complete and recall period	Psychometric properties				Outcomes covered in PD standard set
						Validity	Test–retest reliability	Internal consistency	Sensitivity to change	
Difficulties in emotion regulation scale – 16-Item version (DERS-16)* [33]	16	Patient	> 8	Adults and adolescents	< 5 min	Strong, 0.61–0.93	> 0.85	> 0.92	Good	Emotional dysregulation
Recovering quality of life – 10-Item version (ReQoL10) [34]	10	Patient	9	16+	A few minutes, 1 week recall period	Strong, > 0.8	> 0.85	> 0.87	Weak	Emotional distress, Self-care, Health-related quality of life
Columbia suicide severity rating scale – Screener/Recent Self-report (C-SSRS) [35]	6	Patient	124	All ages	< 5 min, past three months recall period	0.44	N/A	0.94—0.95	Good	Suicide ideation and behaviour
Level of personality functioning scale – Brief form 2.0 (LPFS-BF 2.0) [31]	12	Patient	11	18 + , validated in youth	5 min	Small to moderate	> 0.7	0.89	High	Severity, impulsivity, identity disturbance
Modified overt aggression scale (MOAS)* [36]	4	Clinician	4 (English, Chinese, Italian, French)	18–65, widely used in youth	3 min, recall period 1 week	N/A	> 0.7	0.7–0.9	N/A	Aggression, Self-harm
WHO disability assessment schedule 2.0 – 12-item version (WHODAS12) [37]	12	Patient	> 40	18+	5 min, past 30 days	Adequate	0.83	0.83–0.92	High	Global functioning, social functioning, interpersonal functioning
KIDSCREEN10 index (KIDSCREEN10) [38]	10	Patient	22	8–18	< 5 min	Good	mixed	strong	N/A	Global functioning, social functioning, interpersonal functioning
PROMIS short form v2.0 – Social isolation 4a [39]	4	Patient	4	18+	1 min, in general recall period	N/A	N/A	N/A	N/A	Sense of belonging

*Instruments for measuring optional outcomes

As the number of the outcomes was high, measures that could cover more than one domain were looked for, which could later be complemented by additional instruments. Measures with positive framing of the questions were preferred: this decision was made by the working group following feedback on the content and phrasing from the lived experience representatives.

**Fig. 3:**
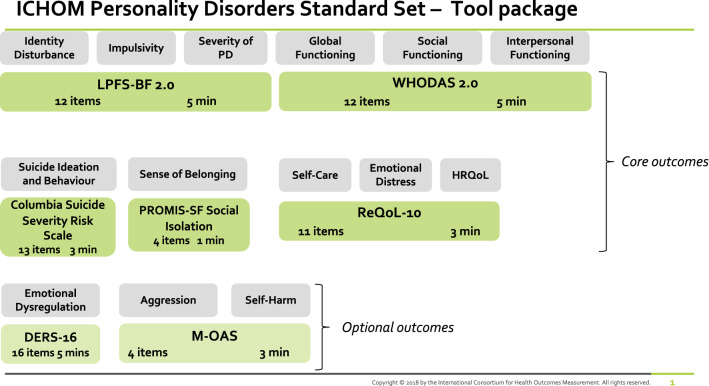
Recommended instrument package with assigned outcomes coverage and timing

Due to a high degree of overlap in the domains that different measures covered, instrument package options were then prepared for voting in the final phase. The group aimed to ensure that the final package of measures would take a person less than 25 min to complete. The final outcomes and the measures are presented in Fig. 3 and Table 3. While most of the outcomes are core, “Emotional Dysregulation”, “Aggression” and “Self-Harm” were included as additional outcome measures for use only in those who experience them. No adequate instrument for “Coping with Past Experiences of Trauma” was identified.

**Table 3:** Outcomes with definitions across outcome domains and corresponding instruments for their measurement, timing for measurement and patient population

Patient population	Outcome	Supporting information	Instrument	Timing
**Mental health**				
All patients	Identity disturbance	An identity disturbance is a deficiency or inability to maintain one or more major components of identity. These components include a sense of continuity over time, emotional commitment to representations of self, role relationships, core values and self-standards, development of a meaningful world view and recognition of one’s place in the world	Level of personality functioning scale – Brief form 2.0 (LPFS-BF 2.0)	Baseline; Ongoing; Six months after discharge; Annually after discharge for two years
	Emotional distress	Subjective experience of a broad range of negative emotions with a frequency and intensity seemingly out of proportion to the situation; emotional lability and poor emotion regulation; negativistic attitudes; low self-esteem and self-confidence; and mistrustfulness	Recovering quality of life – 10-item version (ReQoL10)	
	Emotional dysregulation*	The tendency to display unpredictable, rapidly changing emotions or moods of particularly high intensity	Difficulties in emotion regulation scale – 16-item version (DERS-16)*	
	Suicidal ideation and behaviour	Suicidal ideation, suicidal thoughts or behaviours, suicide attempts, most often accompanied by intense feelings of hopelessness, depression or self-destructive behaviours	Columbia suicide severity rating scale – Screener/Recent-Self-report (C-SSRS)	
**Behaviour**				
All patients	Impulsivity	A predisposition toward rapid, unplanned reactions to internal or external stimuli without regard to the negative consequences of these reactions to the impulsive individual or others	Level of personality functioning scale – Brief form 2.0 (LPFS-BF 2.0)	Baseline; Ongoing; Six months after discharge; Annually after discharge for two years
	Aggression*	Behaviour directed towards another individual with the proximate intent to cause harm	Modified overt aggression scale (MOAS)*	
	Self-harm*	Engaging in self-injury without the intention to die (i.e. scratching, cutting, hitting, burning, picking or head banging)		
**Functioning**				
All patients	Global functioning	An individual’s social, occupational and psychological functioning	WHO disability assessment schedule 2.0 – 12-item version (WHODAS12) Or KIDSCREEN10 index (KIDSCREEN10)	Baseline; Ongoing; Six months after discharge; Annually after discharge for two years
	Social functioning	An individual’s interactions with their environment, the quality of those interactions and the individual’s ability to fulfil their role within such environments as work, social activities and relationships with partners, families and/or friends		
	Interpersonal functioning	Impairments in interpersonal functioning: Empathy: pronounced difficulty understanding impact of own behaviours on others; frequent misinterpretations of others’ motivations and behaviours Intimacy: marked impairments in developing close relationships, associated with mistrust and anxiety	WHO disability assessment schedule 2.0 – 12-item version (WHODAS12) Or KIDSCREEN10 index (KIDSCREEN10)	Baseline; Ongoing; Six months after discharge; Annually after discharge for two years
	Self-care	The ability of individuals to promote and maintain health and well-being, and to cope with illness and disability with or without the support of a health-care provider, including eating well, physical activity and sleep hygiene	Recovering Quality of Life – 10-item version (ReQoL10)	
**Recovery**				
All patients	Sense of belonging	The experience of personal involvement in a system or environment so that persons feel themselves to be an integral part of that system or environment	PROMIS short form v2.0 – Social isolation 4a	Baseline; Ongoing; Six months after discharge; Annually after discharge for two years
	Health-related quality of life	An individual’s perception of their position in life in the context of the culture and value systems in which they live and in relation to their goals, expectations, standards and concerns. It is a broad ranging concept affected in a complex way by the person’s physical health, psychological state, personal beliefs, social relationships and their relationship to salient features of their environment	Recovering quality of life – 10-item version (ReQoL10)	
	Severity of personality disorder	The extent of personality disorder, characterized by problems in functioning of aspects of the self (e.g. identity, self-worth, accuracy of self-view, self-direction), and/or interpersonal dysfunction (e.g. ability to develop and maintain close and mutually satisfying relationships, ability to understand others’ perspectives and to manage conflict in relationships) and is manifest in patterns of cognition, emotional experience, emotional expression and behaviour that are maladaptive	Level of personality functioning scale – Brief form 2.0 (LPFS-BF 2.0)	
**Social environment**				
	Coping with Past Experiences of Trauma**	The use of coping mechanisms in order to cope with historical experiences of trauma, such as abuse, rape and neglect. Can include any behaviour seen as a coping mechanism, including but not limited to as substance misuse and engaging in abusive relationships		

*Optional outcomes

**After a thorough research, the Working Group was not able to identify an adequate outcome measure to capture this outcome. Therefore, the outcome being important to service users, the Standard Set can identify this as a gap in current available outcome measures and make a research recommendation for the future

### Case-mix variables

Case-mix variables are included in the standard set in order to ensure the baseline comparability of treatment populations and intervention factors. ICHOM seeks to extract a minimum set of case-mix variables. Initially, a literature review and extraction from the registries and PD guidelines were performed to identify possible case-mix variables. Case-mix variables were compared against the other ICHOM mental health standard sets, and a harmonized version consisting of demographic and intervention factors was confirmed by the working group (Table 4).

**Table 4:** Summary of demographic, clinical and intervention factors for ICHOM personality disorders standard set

Patient population	Measure	Timing	Data source	
Demographic factors	Year of birth	Baseline	Patient reported	All patients
	Sex			
	Gender identity			
	Sexual orientation			
	Socioeconomic status	Baseline; Transition to adult services; Annually if still in education		
	Work/Education status	Baseline; Annually		
	Housing status			
	Living arrangements			
	Ethnic minority/Marginalization	Baseline		
	Contact with law enforcement	Baseline; Annually		Adult patients; Adolescent patients (where appropriate)
Clinical factors	Comorbidities	Baseline; Annually	Patient reported	All patients
	Hospitalizations		Administrative data	
	Adverse life experiences	Baseline; Transition to adult services	Patient reported	Adult patients; Adolescent patients (where appropriate)
Intervention factors	Intervention setting	Baseline; Annually	Clinical	All patients
	Intervention type			

### Data collection timepoints

**Fig. 4:**
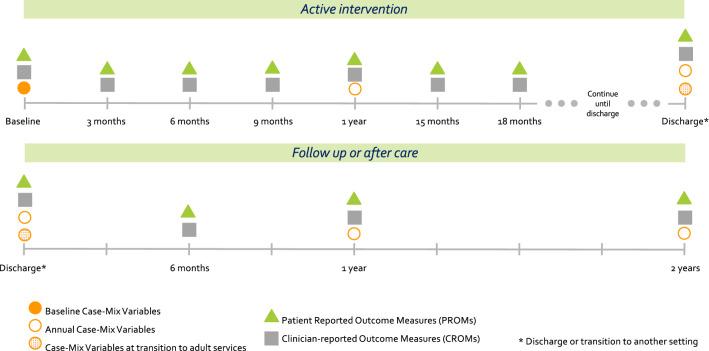
Time guidance on the variables collected from service users and clinicians

Recommended timepoints for the collection of data should be looked at as the minimum requirement for measuring the defined outcomes. The outcome assessment timeline was proposed by the working group to best achieve a balance between the clinically relevant times when outcomes may be expected to change, and the pragmatic concerns in data collection. To harmonize across ICHOM mental health Standard Sets, a meeting between ICHOM mental health working group chairs was held to discuss the timepoints recommendation and suggestions were later voted on by each working group independently. The consensus reached was to recommend assessing outcomes prior to treatment as a baseline, every 3 months in continuous treatment until the discharge and then 6 months after discharge and annually thereafter when not in continuous treatment (Fig. 4).

### Validation process

Seventy responses were received from mental health professionals in 17 countries. The survey was conducted online anonymously, and the respondents used a link to access and complete the survey. The survey was published on ICHOM’s website and shared within a number of newsletters, the mailing lists of which were not disclosed to the authors of this manuscript. No further variables were collected from the respondents. All outcome domains included in the initial recommendation received high endorsement (85% confidence in overall domains) by the professionals in the open review panel. Sixty-three service users responded to the questions in the service users validation survey and the outcomes ‘Aggression’, ‘Identity Disturbance’ and ‘Emotional Dysregulation’ did not reach 70% endorsement. However, these outcomes, as well as the measures proposed to capture them, were highly endorsed by the professional open review panel. All three outcomes were discussed with the working group again, and the proposal was formed to include all the outcomes in the standard set. However, it was decided that ‘Emotional Dysregulation’ and ‘Aggression’ would not be part of the core list of outcomes; the rationale being that not all people with PD experience aggression and emotional dysregulation.

## Discussion

As the case for measuring patient outcomes becomes increasingly accepted by clinicians and decision makers in health care, one of the challenges we are faced with is selecting which measures to use from among the vast array of different instruments that could be used. The ICHOM working group for PD responded to this challenge by selecting and defining a standardized minimum set of outcome measures that would be appropriate to use across different cultural and geographical settings [23]. The included outcomes represent those that matter most to people with PD. The measurement of these outcomes across different environments should help to build better communication between patients and providers. Benchmarking of the results should motivate and empower providers to seek and share good practices and improve care and clinical protocols; payers would be able to clearly see the value of care and make informed decisions on strategic purchase strategies [24]. All the outcomes alongside case-mix variables, timepoints for collection and questionnaires are freely available at the ICHOM website (https://www.ichom.org/standard-sets/).

ICHOM entered the mental health area in 2018. This area depends to an even higher degree on patient-reported outcomes in comparison with some other clinical areas, where clinical readings can describe the outcomes relatively better. Previous research [25] has shown that defining patient-reported outcomes for PD, particularly BPD has many challenges. BPD has heterogeneous clinical features, meaning that patient-reported outcomes should include broad assessment of psychopathology, but at the same time, measure stable as well as more dynamic aspects of the disorder. Social and occupational functioning are especially salient when assessing the outcomes of people with PD, because a number of studies have shown impaired functioning even when mental health improves [26]. Crawford et al. [27] conducted a Delphi study with service providers, services users and academic experts and similarly established that people with a wide range of PD felt that the most important outcome measure that should be assessed was health-related quality of life, followed by mental health and social functioning. Previous attempt [12] to identify core outcome measures in PD that capture quality of life, functioning and symptoms, highlighted that the number of outcomes for BPD is extensive. Above all, this attempt as well as the guidelines on the development of an agreed set of outcomes measures [26] were focused solely on BPD, while the ICHOM recommended standard set is designed for all those with PD and related mental health conditions.

During the whole working process, lasting between October 2018 and June 2020, many scientists, clinicians and service user representatives were included in the formulation of the standard set. All the members discussed the different steps of the work, from defining the scope to the preparation of the final manuscript. Due to the long period of preparing the final set, there was quite a high degree of fluctuation in the project team, as well as in the working group, but all involved expressed their valuable opinion and contributed effectively to the final outcome. The inclusion of the working group members was, however, based on their work recommendations and limited to people from 17 countries. In spite of extensive literature reviews and use of Delphi processes throughout the project, the results might have differed with a different group of participants from different cultural backgrounds.

The primary aim of the standard set is to reflect outcomes that are important to service users. Therefore, including their views and extensive inputs through the whole process is a strength of this work. There were six service user representatives included in the working group and, in the end, 63 users (among them seven carers or parents) reviewed the final version of the standard set, with 94% saying that all important outcomes are captured in the standard set and 96% saying that it would be useful having these outcomes collected. All of the service user representatives came from developed countries and most of them are from Europe.

The process of data collection via suggested questionnaires represents a significant time burden for service users as well as for clinicians. The participants had this in mind and tried to cover all the outcomes in the standard set with as few instruments as possible. Still, the outcomes in the final standard set are measured by eight instruments and the complete collection of all outcomes lasts up to 30 min (including optional instruments). In many countries, the collection of PROMs is still not supported by information communication technology that would enable more efficient collection of data, less reluctance of the stakeholders and automated analysis and results. ICHOM is working on the information of data collection to support the users of the standard sets. In order to promote the use of the standard set in all international environments, the selected outcomes we chose are already translated into many languages. They are available in multiple formats, easily integrated into diverse data collection tools, are computer adaptive and can be used free of charge. A very important issue in mental health standard sets is comorbidity of PDs with other mental health disorders, such as substance use disorders [28], attention-deficit hyperactivity disorder [29] and schizophrenia [30]. Therefore, harmonization of measures across the mental health standard set, as well as among other standard sets that include the same domains, is an important issue, which was taken into account in the process of the selection of measures.

Available evidence suggests that self-assessed measures made by people with PD have high test–retest reliability [31]. However, concerns have been raised about the reliability of self-reported accounts of aggression and other externalizing behaviours [32]. ICHOM aims to establish person-centred outcomes. However, in recognition of the challenges of relying on self-report measures of aggression, a clinician-rated measure, the Modified Overt Aggression Scale, was selected to measure this outcome.

After undertaking a thorough systematic review, the working group was not able to identify an adequate outcome measure to capture the ‘Coping with Past Experiences of Trauma’ outcome. As the outcome is important to service users, the working group identified the lack of an appropriate instrument as a gap in the currently available outcome measures. Future research should be directed toward defining an appropriate instrument to measure this outcome. Various scales measuring similar constructs, such as ‘Posttraumatic growth’ have been looked at in the process and some of them overlap with ‘Coping with Past Experiences of Trauma’. As such, they might be helpful in defining an appropriate measure in the future. Additionally, discriminatory effects among different types of PD through the Standard Set of this ICHOM endeavour need to be studied further.

Furthermore, the standard set is not seen as fixed but should be updated regularly, following new developments in the clinical environment, as well as developments in the health measurement area. The standard set should be seen as a minimal set of outcomes and instruments for their measurement, and further outcomes and measures could be freely added to this set if needed.

## Conclusions

The development of a minimal standard set of value-based service user-centred outcome measures in PD should lead to higher value of care, and better outcomes of care, for people with PD all across the world. Widespread use of these measures will lead to benchmarking and exchange of good practices, to greater inclusion of service users in care processes, and to better communication between clinicians and service users. It will also provide the payer with evidence that could serve as a basis for informed decision making on allocation of funds.
